# Evaluation of antihyperglycemic and antinociceptive activity of *Xanthium indicum* stem extract in Swiss albino mice

**DOI:** 10.1186/1472-6882-13-296

**Published:** 2013-10-30

**Authors:** Md Ezazul Haque, Shahnaz Rahman, Mohammed Rahmatullah, Rownak Jahan

**Affiliations:** 1 Department of Biotechnology and Genetic Engineering, University of Development Alternative, Dhanmondi, Dhaka 1209, Bangladesh; 2 Faculty of Life Sciences, University of Development Alternative, House No. 78, Road No. 11A (new), Dhanmondi, Dhaka 1209, Bangladesh

**Keywords:** Antihyperglycemic, *Xanthium indicum*, Glucose tolerance, Antinociceptive, Asteraceae

## Abstract

**Background:**

*Xanthium indicum* stem is used in folk medicine of Bangladesh to control sugar in diabetic patients and to alleviate pain. The objective of the study was to evaluate antihyperglycemic and antinociceptive activity of methanolic extract of *Xanthium indicum* stems (XISE) in mice.

**Methods:**

Antihyperglycemic activity was measured by oral glucose tolerance tests in glucose-loaded Swiss albino mice. Antinociceptive activity was determined by observed decreases in abdominal constrictions in acetic acid-induced gastric pain model in mice.

**Results:**

The methanol extract of stems showed dose-dependent and statistically significant antihyperglycemic activity at doses of 50, 100, 200 and 400 mg per kg body weight (p values, respectively, < than 0.01, 0.01, 0.005, and 0.01). Highest reduction in blood glucose level (31.2%) was observed with the highest dose (400 mg) of the extract. A standard antihyperglycemic drug, glibenclamide, reduced blood glucose levels by 46.2%, when administered at a dose of 10 mg per kg body weight. In antinociceptive activity tests, the extract when administered at the afore-mentioned four doses, reduced the number of abdominal constrictions in mice, respectively, by 41.7, 50.0, 54.2, and 61.0%. In comparison, a standard antinociceptive drug, aspirin, when administered at a dose of 200 mg per kg body weight, reduced the number of abdominal constrictions by 37.5%.

**Conclusion:**

The experimental results obtained in the present study validate the use of *X. indicum* stems in folk medicines of Bangladesh to lower blood sugar in diabetic patients and to alleviate pain.

## Background

*Xanthium indicum* J. Koenig (Asteraceae) is a coarse annual plant, which grows to about a meter high and is found in the wild and fallow lands of Bangladesh. It is known as 'burweed’ in English and 'ghagra’ in Bengali. Ethnomedicinal uses in Bangladesh include using the plant to control blood sugar in diabetic patients and for treatment of rheumatic pain [1]. The plant is used by the Lohit community of Arunachal Pradesh, India for treatment of inflammation-related diseases [2]. A recent report has demonstrated anti-bacterial and cytoxic activities in methanolic extract of the plant [3].

Medicinal plants used by indigenous communities and in traditional medicinal systems have always been the source of many modern allopathic dugs [4]. As such, the objective of the present study was to evaluate the antihyperglycemic and antinociceptive potential of *X. indicum* stems in oral glucose tolerance test (OGTT) conducted with glucose-loaded Swiss albino mice, and in antinociceptive tests conducted with intraperitoneally injected acetic acid-induced gastric pain model in mice.

## Methods

Stems of *X. indicum* were collected during March 2013 from Shabdi in Narayanganj district, Bangladesh and taxonomically identified at the Bangladesh National Herbarium (Accession Number 35,026). Stems were cut into small pieces, air-dried in the shade, and 30 g of dried and powdered stems was extracted with methanol (w:v ratio of 1:7, final weight of the extract 1.73 g). Glibenclamide, aspirin, and glucose were obtained from Square Pharmaceuticals Ltd., Bangladesh. All other chemicals were of analytical grade.

In the present study, Swiss albino mice (male), which weighed between 18–22 g were used. The animals were obtained from International Centre for Diarrheal Disease Research, Bangladesh (ICDDR,B). The animals were acclimatized for three days prior to actual experiments. The study was conducted following approval by the Institutional Animal Ethical Committee of University of Development Alternative, Dhaka, Bangladesh.

Glucose tolerance property of extract was determined as per the procedure previously described by Joy and Kuttan [5] with minor modifications. Briefly, fasted mice were grouped into six groups of six mice each. The various groups received different treatments like Group 1 received vehicle (1% Tween 80 in water, 10 ml/kg body weight) and served as control, Group 2 received standard drug (glibenclamide, 10 mg/kg body weight). Groups 3–6 received extract at doses of 50, 100, 200 and 400 mg per kg body weight. All substances were orally administered. Following a period of one hour, all mice were orally administered 2 g glucose/kg of body weight. Blood samples were collected 120 minutes after the glucose administration through puncturing heart. Blood glucose levels were measured by glucose oxidase method [6].

Antinociceptive activity of the extract was examined using previously described procedures [7]. Briefly, mice were divided into six groups of five mice each. Group 1 served as control and was administered vehicle only. Group 2 was orally administered the standard antinociceptive drug aspirin at a dose of 200 mg per kg body weight. Groups 3–6 were administered extract at doses of 50, 100, 200 and 400 mg per kg body weight, respectively. Following a period of 60 minutes after oral administration of standard drug or extract, all mice were intraperitoneally injected with 1% acetic acid at a dose of 10 ml per kg body weight. A period of 5 minutes was given to each animal to ensure bio-availability of acetic acid [8], following which period, the number of abdominal constrictions was counted for 10 min.

Experimental values are expressed as mean ± SEM. Independent Sample *t*-test was carried out for statistical comparison. Statistical significance was considered to be indicated by a p value < 0.05 in all cases [8].

## Results and discussion

Administration of the extract to glucose-loaded mice led to dose-dependent and statistically significant reductions in the levels of blood glucose levels. The results are shown in Table 1. At doses of 50, 100, 200 and 400 mg extract per kg body weight, blood glucose levels in experimental mice were, respectively, 74.1, 73.5, 71.8, and 68.8% compared to blood glucose level in control mice (100%). Thus the percent reductions in blood glucose levels in experimental mice administered with the four doses of the extract were respectively, 25.9, 26.5, 28.2, and 31.2. A standard antihyperglycemic drug, glibenclamide, when administered at a dose of 10 mg/kg body weight reduced blood sugar levels by 46.2%. Thus the extract, at the doses tested, while being not as potent as glibenclamide, still demonstrated considerable antihyperglycemic activity.

**Table 1:** **Effect of methanol extract of *X. indicum *** **stems (XISE) on blood glucose level in hyperglycemic mice following 120 minutes of glucose loading**

**Treatment**	**Dose (mg/kg body weight)**	**Blood glucose level (mmol/l)**	**% lowering of blood glucose level**
Control (Group 1)	10 ml	5.67 ± 0.32	**-**
Glibenclamide (Group 2)	10 mg	3.05 ± 0.06	46.2*
(XISE)	50 mg	4.20 ± 0.27	25.9*
(XISE)	100 mg	4.17 ± 0.22	26.5*
(XISE)	200 mg	4.07 ± 0.18	28.2*
(XISE)	400 mg	3.90 ± 0.31	31.2*

All administrations were made orally. Values represented as mean ± SEM, (n = 6); ^*^P < 0.05; significant compared to hyperglycemic control animals.

As far as the mechanism of the observed antihyperglycemic effect is concerned, it is possible that the extract may be acting through potentiating pancreatic insulin secretion, or through increasing glucose uptake [5]. Such mechanisms have also been proposed for root extract of *Helicteres isora *[9].

In antinociceptive activity tests, the extract at doses of 50, 100, 200 and 400 mg per kg body weight reduced the number of abdominal constrictions induced by intraperitoneal acetic acid injection in mice, respectively, by 41.7, 50.0, 54.2, and 61.0%. A standard antinociceptive drug, aspirin, when administered at a dose of 200 mg per kg body weight, reduced the number of abdominal constrictions by 37.5%. Thus the extract, even at the lowest dose tested, demonstrated more potent antinociceptive activity than aspirin. The results are shown in Table 2.

**Table 2:** **Antinociceptive effect of crude methanol extract of *X. indicum *** **stems (XISE) in the acetic acid-induced gastric pain model mice**

**Treatment**	**Dose (mg/kg body weight)**	**Mean number of abdominal constrictions**	**% inhibition**
Control (Group 1)	10 ml	4.8 ± 0.20	-
Aspirin (Group 2)	200 mg	3.0 ± 0.55	37.5*
(XISE)	50 mg	2.8 ± 0.86	41.7*
(XISE)	100 mg	2.4 ± 1.08	50.0*
(XISE)	200 mg	2.2 ± 1.16	54.2*
(XISE)	400 mg	2.0 ± 0.84	61.0*

All administrations (aspirin and extract) were made orally. Values represented as mean ± SEM, (n = 5); *P < 0.05; significant compared to control.

As mentioned before, the plant has been reported to have ethnomedicinal uses for treatment of inflammatory conditions [2], which is almost always accompanied with pain. It has been reported that acetic acid acts indirectly by inducing the release of mediators like prostaglandin E2, as well as lipooxygenase products [10]. Production of prostaglandins [mainly prostacyclines (PGI_2_) and prostaglandin- (PG-E)] has been shown to be responsible for excitation of Aδ-nerve fibers, leading to the sensation of pain [11,12]. As such, the antinociceptive activity exhibited by extract may be due to the extract’s ability to block synthesis of prostaglandins. This, in turn, may be mediated through inhibition of cyclooxygenase and/or lipooxygenase activities. A similar mechanism has been proposed for antinociceptive activity of *Ficus deltoidea* aqueous extract in acetic acid-induced gastric pain model [10]. That the extract of the plant proved to be more potent than aspirin in demonstration of antinociceptive activity can prove beneficial for at least two reasons. First, the plant can serve particularly the poor population of Bangladesh with a cheap and readily available source of a pain-killing medication. Second, the extract may contain phytochemical constituent(s), which may prove better than aspirin in alleviating pain and causing less side-effect, and as such, can be a more efficacious allopathic medicine. Notably, over-dosage or prolonged use of aspirin can cause gastric ulcerations.

It is also to be noted that antinociceptive activity of leaf extract of the plant has been reported before [13,14], but this is the first report on antihyperglycemic and antinociceptive activity of stem extract of the plant. A point to be mentioned in this regard is that the leaf extract of the plant, when administered at doses of 50, 100, 200 and 400 mg per kg body weight was reported to reduce the number of acetic acid-induced abdominal constrictions in mice by 31.8, 36.6, 41.2, and 42.9%, respectively [13]. The stem extract, on the other hand of the same plant, at the afore-mentioned four doses, reduced the number of acetic acid-induced abdominal constrictions by 41.7, 50.0, 54.2, and 61.0%, respectively. Thus, the stem extract proved to be more potent than the leaf extract, and may contain greater concentrations of similar analgesic component(s), or more potent but different analgesic component(s) than the leaf extract. It is to be noted that a hypoglycemic agent, carboxyatractyloside, has been isolated from a related species, *X. strumarium *[15]. *X. strumarium* extract also reportedly demonstrated antinociceptive property, which was attributed to presence of caffeoylquinic acids in the extract [16]. It is possible that similar antihyperglycemic and antinociceptive constituents may also be present in *X. indicum*.

## Conclusion

The results validate the folk medicinal use of stems of *X. indicum* to reduce high blood glucose levels in diabetic patients and to alleviate pain. From that view point, the extract merits further scientific attention for further isolation and identification of the responsible bioactive component(s). Such identification was not done in this preliminary report, but is currently undergoing in our laboratory.

## Competing interests

The authors declare that they have no competing interests.

## Authors’ contributions

MEH and SR collected the plant, did the extraction, and performed the experiments under the supervision of MR. MR wrote the manuscript draft, which was read and edited by all authors. All authors read and approved the final version of the manuscript.

## Pre-publication history

The pre-publication history for this paper can be accessed here:

http://www.biomedcentral.com/1472-6882/13/296/prepub
